# Revisiting Physiological and Operative Severity Score for the Enumeration of Mortality and Morbidity (POSSUM) and Portsmouth-POSSUM (P-POSSUM) Scores: Are They Valid in Cases of Ileal Perforation?

**DOI:** 10.7759/cureus.65733

**Published:** 2024-07-30

**Authors:** Saikrishna Eswaravaka, Chirantan Suhrid, Bhavya Rao, Sundaresh Prabhakar, Jayashri Pandya

**Affiliations:** 1 Surgical Gastroenterology, All India Institute of Medical Sciences, Jodhpur, Jodhpur, IND; 2 General Surgery, Topiwala National Medical College & B. Y. L. Nair Charitable Hospital, Mumbai, IND; 3 Surgical Oncology, King George's Medical University, Lucknow, IND

**Keywords:** medical school education, scoring systems, multiple ileal perforations, p-possum score, possum

## Abstract

Introduction

Ileal perforation due to typhoid is common in tropical countries, and the ensuing secondary peritonitis is treated by resuscitation and surgery. The Physiological and Operative Severity Score for the Enumeration of Mortality and Morbidity (POSSUM) was developed to predict postoperative outcomes to overcome systemic obstacles in any healthcare setup and is considered fairly accurate. The Portsmouth-POSSUM (P-POSSUM) score was developed as a corrective scoring system for overestimations made by the POSSUM score vis-à-vis mortality. Our study aimed to examine the validity of these two scores in the postoperative prediction of surgical outcomes in patients with ileal perforation.

Materials and methods

An observational study involving 40 patients diagnosed with ileal perforations was undertaken over 18 months. The postoperative outcome for each patient was calculated as per the POSSUM and P-POSSUM parameters. Statistical analysis was done using SPSS (IBM Corp., Armonk, NY) and the results were tabulated.

Results

We found that age, gender, respiratory dysfunction, propensity for multiple surgeries, duration of surgery, co-morbidities, underlying malignancy, and systolic blood pressure played a significant role in determining postoperative outcomes. Haemoglobin, potassium, and urea levels were also found to be significantly associated with outcome. Cardiac signs, pulse, white blood cell count, Glasgow Coma Scale score, sodium, and electrocardiography, part of the physiological score parameters, were found to be insignificant in the prediction of postoperative outcomes. Among the intraoperative parameters, peritoneal soiling was found to be insignificant.

Conclusion

Some parameters inherent to POSSUM and P-POSSUM calculations appear to bear no statistical significance to the final score, highlighting that these need to be revisited and perhaps modified to further simplify the calculation. The POSSUM score is an excellent predictor of postoperative morbidity and mortality in ileal perforation patients but is of questionable reliability due to its tendency to overestimate them. P-POSSUM has a better predictive power of postoperative mortality by correcting POSSUM mortality overestimation.

## Introduction

Ileal perforation is common in tropical countries, the most common cause being typhoid fever. In Western countries, the causes range from malignancy to mechanical causes. Ileal perforation is a potentially fatal complication of enteric fever, which usually occurs by the third week of the disease when inadequately treated. The pathology behind this perforation in the terminal ileum, generally preceded by haemorrhage, appears to be necrosis of Peyer patches, which are maximum in density in this region of the bowel. Overall, mortality due to ileal perforation secondary to typhoid fever has declined worldwide because of the use of antibiotics, improved surgical care, and supportive therapy [1].

The incidence of perforation in typhoid fever has been reported to be 0.8% to 18%. Tuberculosis accounts for 5-9% of all small intestinal perforations in India and is the second most common cause. The standard of care for secondary peritonitis due to hollow viscus perforation is resuscitation, followed by emergency exploratory laparotomy. The methods of the management for ileal perforations include primary closure, resection and anastomosis of the small gut, limited right hemicolectomy, and diverting stoma, depending on the site of perforation, number of perforations, severity of peritonitis, and general condition of the patient. Early diagnosis, prompt and adequate resuscitation, and early treatment avoid the need for extensive surgery [2].

In general, as patients are in an emergency situation, they often require ileostomy as a lifesaving measure. However, in Western countries, indications for ileostomy are altogether different and include inflammatory bowel disease, familial adenomatous polyposis, colorectal cancer, pelvic sepsis, trauma, diverticulitis, fistula, ischemic bowel disease, radiation enteritis, faecal incontinence, and paraplegia. Ileostomy serves the purpose of diversion, decompression, and exteriorization. Primary ileostomy has been found to be superior to other surgical procedures as far as morbidity and mortality are concerned [2]. Factors influencing operative outcomes in developing countries are distinct from those affecting clinical and recovery parameters in developed countries due to variance in physiological, economic, and socio-cultural aspects [1].

Scoring systems are indispensable for the triage of critically ill patients. Comparing crude mortality and morbidity rates is a fallacious exercise, owing to variations in the general health of a local population and variable presentations. Most systems provide a realistic expectation of the patient’s outcome and can be used to suggest the allocation of resources in resource-deficient settings, like ours [3].

When scoring systems are risk-adjusted, they incorporate the patient’s diagnosis along with the management protocol being followed for treatment. Like the Acute Physiology and Chronic Health Evaluation (APACHE) score that is used to assess the severity of illness of patients in intensive care units, the Physiological and Operative Severity Score for the Enumeration of Mortality and Morbidity (POSSUM) score was developed to predict postoperative morbidity and mortality and is considered fairly accurate without being cumbersome [3].

POSSUM has been widely used since it was first introduced by Copeland et al. in 1991 for its simplicity, with the added advantage of considering operative findings while calculating it [4]. The POSSUM score was developed as an attempt to quantify the quality of surgical care and to allow comparison among various surgeons, units, hospitals, and regions. Initially, 62 factors were examined as predictors of postoperative morbidity and mortality. Using mathematical prediction and multi-variant analysis, a set of 12 physiological and six operative parameters was identified to be the most powerful predictor of morbidity and mortality. While the other factors play a role in outcome prediction, they were found not to have any predictive powers. A variant of the POSSUM score, Portsmouth-POSSUM (P-POSSUM), uses the same 18 parameters but, to enhance predictive power, the numerical constants were modified. Both these scores in the last decade have been applied in elective and emergency surgeries for risk assessment [5].

The operative protocol adopted for the management of surgical pathology is chosen with the intention of avoiding mishaps and/or complications. To validate the POSSUM and its variant, the P-POSSUM score, as being one that can be recorded easily and reproduced satisfactorily by resident staff, we undertook this study to assess its effectiveness in the prediction of morbidity and mortality after surgery for ileal perforation.

## Materials and methods

An observational study was done at our tertiary care centre including 40 patients over an 18-month period to assess the effectiveness of the POSSUM score for the prediction of morbidity and mortality after surgery for ileal perforation.

All cases of ileal perforation undergoing surgery in our tertiary care centre irrespective of indication (except traumatic injury to the abdomen) and aged more than 18 years were included in the study. Pregnant patients and those suffering from traumatic ileal perforation were excluded from this study.

The sample size was calculated using the formula: n = [z^2^p(1-p)]/d^2^. Where z = table value of alpha error from standard normal distribution table (0.95); power (p) = 80%; and precision error of estimation (d) = 0.65.

n = [0.95 x 0.95 x 0.8 (0.2)] / 0.65 x 0.65 = 33.18.

Hence, a sample size of 40 patients was selected for the study.

All patients were thoroughly evaluated with detailed history, clinical examination, and blood investigations including complete blood counts, blood urea, X-ray of the chest in erect position, Widal test, and blood culture. The procedure was explained to the patients and written consent was taken for surgery. All cases were managed with intravenous fluids for resuscitation, a nasogastric tube for gut decompression, urethral catheterization to monitor urine output, third-generation cephalosporins, and analgesics. After initial resuscitation in the emergency department, patients underwent emergency laparotomy through midline incision. The intraoperative findings, namely, site, number, and size of perforations, extent of peritonitis, condition of gut, status of lymph nodes, and mesentery, were recorded and thorough peritoneal lavage was done. Loop ileostomy or resection and anastomosis were done as per the standard methods. Patients were monitored postoperatively and their histopathology reports were traced and registered.

Predicted morbidity and mortality scores were calculated based on physiologic and operatives scores using the POSSUM equation as follows:

(1) POSSUM equation for predicting morbidity: Logn R/(1-R) = -5.91 + (0.16 × physiological score) + (0.19 × operative severity score).

(2) POSSUM equation for predicted mortality: Logn R/(1-R) = -7.04 + (0.13 × physiological score) + (0.16 × operative severity score).

(3) P-POSSUM equation for predicting mortality: Logn R/(1-R) = −9.37 + (0.19 × physiological score) + (0.15 × operative severity score).

The actual number of patients having any morbidity and mortality was recorded and compared with the expected number as calculated using POSSUM and P-POSSUM.

Quantitative data are presented with the help of mean and standard deviation. Comparison among the study groups is done with the help of an unpaired t-test as per the results of the normality test. Qualitative data are presented with the help of frequency and percentage tables. Association among the study groups is assessed with the help of the Fisher's test, Student's t-test, and chi-square test. A p-value less than 0.05 was taken as significant. Results were graphically represented where deemed necessary.

## Results

In our study, gender was found to be significant in the prediction of outcomes (p < 0.05). The distribution of males and females was 29 and 11, respectively, as shown in Table 1.

**Table 1:** Distribution of gender "S" stands for "significant" as the p-value obtained is <0.05.

	Survival	
Sex	Yes	No
Female	7	4
Male	26	3
P-value	0.046	S

The choice of surgery was found to be statistically significant in the prediction of survival. Table 2 shows that most of the patients who survived had undergone primary closure of ileal perforation (n = 21) while most of the patients who did not survive had undergone resection anastomosis (n = 4).

**Table 2:** Association of surgery and outcome "S" stands for "significant" as the p-value obtained is <0.05.

	Survival	
Surgery	Yes	No
Double barrel stoma	6	3
Primary closure	21	0
Resection anastomosis	6	4
P-value	0.011	S

According to clinical findings pertaining to the respiratory system, three patients with respiratory dysfunction in the form of dyspnoea and one patient who suffered from mild chronic obstructive pulmonary disease (COPD) did not survive, as evidenced in Table 3. A statistically significant correlation was noted between respiratory status and survival.

**Table 3:** Co-relation of respiratory dysfunction with mortality "S" stands for "significant" as the p-value obtained is <0.05.

	Survival	
Respiratory system	Yes	No
Dyspnoea	0	3
Mild chronic obstructive pulmonary disease (COPD)	1	1
Within normal limits (WNL)	32	3
P-value	0.001	S

We observed a significant association between the number of procedures and mortality. One patient who did not survive had undergone multiple procedures, as accounted for in Table 4.

**Table 4:** Distribution according to the number of procedures "S" stands for "significant" as the p-value obtained is <0.05.

	Survival	
Multiple procedures	Yes	No
1	33	6
2	0	1
P-value	0.028	S

Table 5 shows that a statistically significant association was found between the duration of surgery and outcomes. The majority of patients who had surgeries lasting less than two hours survived (33 out of 40).

**Table 5:** Association of duration of surgery with outcome "S" stands for "significant" as the p-value obtained is <0.05.

	Survival	
Duration of surgery	Yes	No
>2 hours	0	3
<2 hours	33	4
P-value	0.00	S

Comorbidities that the patient suffered from appeared to play a statistically significant role in the outcome after surgery (p < 0.05). Table 6 shows that the majority of our patients did not suffer from any comorbidities. Among the tabulated comorbidities, wound infection accounted for the most number of cases (n = 11). Pneumonia was seen in seven patients. Urinary tract infection, wound dehiscence, and respiratory failure were the comorbid conditions that five patients were affected by. Renal failure was identified in three patients while pyrexia of unknown origin and resection anastomosis (RA) leak occurred in two patients each. One patient each was found to suffer from thrombosis and cardiac failure.

**Table 6:** Prevalence of morbidity among patients

Morbidity	Number of cases
Pneumonia	7
Pyrexia of unknown origin	2
Thrombosis	1
Urinary tract infection	5
Wound infection	11
Wound dehiscence	5
Renal failure	3
Cardiac failure	1
Resection anastomosis leak	2
Respiratory failure	5
None	19
P-value	0.0092

The association of age with outcome showed a significant association (p = 0.032). A significant association was also found between recorded systolic blood pressure and surgical outcome for the patient where the p-value was found to be 0.021.

Haemoglobin (Hb) levels recorded at the time of admission, as tabulated in Table 7, were found to have a significant association with outcome post surgery (p < 0.05). Among those who survived, mean Hb levels were found to be 13.24 g /dL with a standard deviation of 2.23. The highest value obtained among those who survived was 18.1 g/dL and the lowest was 8.2 g/dL. In contrast, the mean Hb level among those who succumbed was found to be 9.11 g/dL with a standard deviation of 2.34, and the highest and lowest values of Hb in this population were 12.7 g/dL and 6.3 g/dL, respectively.

**Table 7:** Association of haemoglobin (Hb) with outcome "S" stands for "significant" as the p-value obtained is <0.05.

Hb	Mean (in g/dL)	Std. deviation	Minimum (in g/dL)	Maximum (in g/dL)	P-value
Succumbed	9.11	2.34	6.3	12.7	0.001
Survived	13.24	2.23	8.2	18.1	S

Our study found that WBC levels at the time of admission played no significant role in predicting the postoperative outcome of patients.

Serum potassium level was seen to be significantly associated with postoperative outcome (p < 0.05), with a mean value of 4.1 mmol/L among those who survived. The standard deviation was computed to be 0.63 as well. Among those who succumbed, the mean serum potassium level was 3.6 mmol/L, with a standard deviation of 0.41. The highest potassium value seen among those who succumbed was 4.2 mEq/L, almost coinciding with the mean value among those who survived (0.41 mmol/L), as shown in Table 8.

**Table 8:** Association of potassium with outcome "S" stands for "significant" as the p-value obtained is <0.05.

K	Mean (in mmol/L)	Std. deviation	Minimum (in mmol/L)	Maximum (in mmol/L)	P-value
Succumbed	3.6	0.41	3	4.2	0.05
Survived	4.10	0.63	2.7	5.4	S

We found that sodium levels were insignificant in predicting postoperative outcomes as the p-value was found to be >0.05.

Table 9 shows that urea levels were found to be significantly associated with outcome as the p-value was 0.001. A mean value of 16.27 mg/dL was calculated among those who survived with a standard deviation of 9.44. Among those who did not survive, the mean serum urea level was found to be 39.14 mg/dL with a standard deviation of 30.02. The highest level of serum urea was seen to be 80 mg/dL and the lowest was 7 mg/dL.

**Table 9:** Association of urea with outcome "S" stands for "significant" as the p-value obtained is <0.05.

Urea	Mean (in mg/dL)	Std. deviation	Minimum (in mg/dL)	Maximum (in mg/dL)	P-value
Succumbed	39.14	30.02	7	80	0.001
Survived	16.27	9.44	5	37	S

Total blood loss during surgery for ileal perforation was also found to be significantly associated with outcome (p = 0.002).

We observed a statistically significant association between malignancy and postoperative outcome (p < 0.04), as per Table 10. Among the patients who succumbed, we found that six patients had no malignancy and one patient had malignancy associated with lymphadenopathy.

**Table 10:** Association of malignancy with outcome "S" stands for "significant" as the p-value obtained is <0.05.

	Survival	
Malignancy	Yes	No
No	32	6
Yes	1	0
Yes, with lymphadenopathy	0	1
P-value	0.04	S

After using the formulae to calculate POSSUM morbidity and mortality scores, we found that the mean morbidity and mortality scores in the patients who had acceptable postoperative outcomes were 73.78 (standard deviation of 14.84) and 26.5 (standard deviation of 14.14), respectively. The mean scores for patients who had adverse postoperative results were found to be 95.45 (standard deviation of 6.84) and 69.28 (standard deviation of 21.19), respectively. These values were found to be statistically significant as the p-value was found to be <0.05, as shown in Table 11.

**Table 11:** Association of POSSUM morbidity and POSSUM mortality with outcome "S" stands for "significant" as the p-value obtained is <0.05. POSSUM: Physiological and Operative Severity Score for the Enumeration of Mortality and Morbidity.

POSSUM morbidity	Mean	Std. deviation	Minimum	Maximum	P-value
Succumbed	95.45	6.84	80.4	99.1	0.001
Survived	73.78	14.84	45.3	94.8	S
POSSUM mortality	Mean	Std. deviation	Minimum	Maximum	P-value
Succumbed	69.28	21.19	27.3	84.7	0.00
Survived	26.5	14.14	9.3	56.2	S

A comparison of the expected mortality rate using the POSSUM score to the observed actual mortality showed a ratio of less than 1, denoting that the POSSUM score overestimates mortality, as shown in Table 12. For example, in all cases where there was a calculated expectation of mortality, the actual observed mortality numbers were always lesser than the expectation.

**Table 12:** Comparison of expected mortality using POSSUM score with observed mortality POSSUM: Physiological and Operative Severity Score for the Enumeration of Mortality and Morbidity; O/E ratio: observed-to-expected ratio.

Predicted mortality rate	Mean predicted mortality rate	Expected number of death	Observed number of death	O/E ratio
Less than 10	15.2	1	0	0
10 to 20	16.5	1	0	0
20 to 30	24.3	2	1	0.5
30 to 40	0	2	0	0
40 to 50	0	0	0	0
50 to 60	57.1	3	1	0.33
60 to 70	0	0	0	0
70 to 80	76.4	5	3	0.6
80 to 90	84.5	5	2	0.4
90 to 100	0	0	0	0

Table 13 shows a comparison of the expected rate of complications (morbidity) using the POSSUM score to the observed incidence of complications (morbidity) showing the ratio to be less than 1, denoting that the POSSUM score overestimates the incidence of morbidity.

**Table 13:** Comparison of expected morbidity using POSSUM score with observed morbidity POSSUM: Physiological and Operative Severity Score for the Enumeration of Mortality and Morbidity; O/E ratio: observed-to-expected ratio.

Predicted morbidity rate	Mean predicted morbidity rate	Expected number of complications	Observed number of complications	O/E ratio
Less than 10	0	0	0	0
10 to 20	0	0	0	0
20 to 30	0	0	0	0
30 to 40	0	0	0	0
40 to 50	42.4	1	0	0
50 to 60	55.5	2	0	0
60 to 70	63.7	1	0	0
70 to 80	74.8	6	2	0.3
80 to 90	86.2	6	2	0.3
90 to 100	94.8	6	4	0.7

## Discussion

The basic tenet in health care is to provide quality health care with minimal adverse outcomes. A comparison of adverse outcome rates is necessary to assess the adequacy of care and to evolve new strategies for better outcomes. The accurate prediction of outcomes after high-risk procedures can detect early postoperative complications, and early referral to a higher medical facility. Better planning and precision can improve individual prognosis and it can help in strengthening India’s tier-based referral service.

POSSUM was developed to overcome the shortcomings of crude mortality rate-based comparison. Postoperative complications and death may result depending on three major factors: the quality of the surgical team, the patient’s physiological status, and the degree of surgical stress [5]. However, POSSUM has to be correlated to the general condition of the local population for it to be effective [6]. This is important for patients in developing countries like India where the general health of the population is variable and presentation is frequently delayed [7,3].

While one of the limitations of the study may be perceived to be the small sample size and correspondingly small number of outcomes, one must note that the sample that was chosen was as per protocol, after applying statistically relevant equations, so that a relatively small number may still be used as a surrogate for the general population. Thus, the application of tests of significance that have yielded our results cannot be disregarded as insignificant. Some may also argue that the inherent limitation of the study being its small sample size can create a higher risk of overfitting. While this may be true, most of the observations that have been made in this study have been justified with an adequate review of existing literature, either opposing or allying with our findings and each was given an explanation in the discussion that follows and thus, must not be dismissed as being of no importance.

Considering that intraoperative parameters are required for the calculation of the POSSUM and P-POSSUM scores, their usage may also be viewed as cumbersome, particularly in matters concerning referral to higher centres. While we acknowledge that these perceived limitations may hold true, surgical outcomes are unpredictable and in our referral-based system, peripheral hospitals may not have the wherewithal to manage complicated patients. Thus, we still advocate for the use of these scores, especially in far-flung areas, so that they can predict bad outcomes before they occur and put the referral system to good use.

We found that the majority of the patients presenting with ileal perforation were males, accounting for 72.5% of our population. Males were more susceptible to ileal perforations, as evidenced by the study carried out by Poornima et al., wherein 81.25% of their study population was also found to be male [8]. This is important to note as gender is significantly correlated with the outcome of surgery, making it a significant contributor to the predictions made by the POSSUM score on postoperative outcomes.

Reviewing the literature, we found that most ileal perforations are surgically managed by primary closure when compared to other modalities of management. For example, in the study carried out by Ilahi et al., approximately 75% of all enteric perforations, including ileal perforations, were managed by primary closure [9]. As the surgical approach chosen for management is significantly associated with postoperative outcomes, its role in POSSUM prediction is stark. Considering that other parameters like intraoperative blood loss and operative severity are also significant indicators of postoperative outcomes, it is only logical that primary repair of the perforation is most often chosen to decrease the intraoperative adverse effects, increasing the chances of better patient outcomes.

In the study carried out by Kassahun and colleagues, the impact of preoperative respiratory diseases, particularly COPD, on postoperative outcomes was studied and the results suggested that COPD was significantly associated with an enhanced risk of postoperative pulmonary complications, dependence on invasive ventilation, and thromboembolisms. The study also found that in-hospital mortality had no association with COPD [10]. Our study also found that the respiratory component of POSSUM was significantly correlated with postoperative outcomes. Thus, the validity of including respiratory illness in the calculation of POSSUM appears justified, albeit only in the prediction of morbidity and not mortality.

Multiple procedures being performed for ileal perforation were also found to be statistically significant vis-à-vis postoperative outcomes. Our study included one patient for whom more than one procedure was done for the management of ileal perforation and that patient did not survive. Higher time spent in the operation theatre and the ensuing increased blood loss, both of which are significantly associated with postoperative outcomes, explain this. This implies that a longer duration of surgery negatively affects postoperative outcomes, as evidenced by our study as well. For example, the study conducted by Bohnen et al. shows that intraoperative adverse events, which increase the duration of intraoperative time, substantially increase postoperative morbidity and mortality [11].

Although not a part of the POSSUM score parameters, we found that comorbidities that a patient suffers from play a significant role in predicting their postoperative outcome. Out of the 40 patients included in our study, 19 patients did not suffer from any comorbidities and hence, had better postoperative outcomes.

Hb levels at the time of admission were found to be significantly associated with postoperative outcomes and are one of the constituent parameters of the POSSUM calculation. We found that patients who had a Hb level of 13.24 +/- 2.23 g/dL survived and those with a mean Hb level of 9.11 g/dL did not survive. The inclusion of Hb levels in the POSSUM score appears justified as multiple studies show how preoperative anaemia plays a significant role in the postoperative outcome of patients undergoing surgery. As aptly put in Michailidou et al.’s study on patients suffering from inflammatory bowel disease, lowered levels of preoperative Hb serve as an independent predictor, irrespective of its inclusion in other scoring systems of postoperative complications and morbidity in cases of abdominal surgery [12].

Sodium and potassium levels are a significant part of the POSSUM score and contribute towards identifying postoperative outcomes. We found that a significant association was seen only with preoperative potassium levels. Those with a mean potassium level of 3.6 mmol/L had a higher likelihood of adverse postoperative outcomes than those with a mean potassium level of 4.1 mmol/L at the time of admission. Multiple studies show similar outcomes for hypokalaemic patients, like the study carried out by Arora et al., wherein they found that the risk of mortality within 30 days of surgery was 13.6% in patients with hypokalaemia when compared to their normokalemic counterparts who had a risk of 4.9%. What is also striking is that our discovery of patients with a higher risk of adverse outcomes when their potassium levels were 3.6 mmol/L fits into their definition for hypokalaemia, i.e., <4 mmol/L [13].

Among other predictors, urea was found to be significantly associated with postoperative outcomes in our study, validating its use in the POSSUM calculation. A mean urea level of 16.27 mg/dL was found to have a significantly better postoperative outcome than a mean value of 39.14 mg/dL in our study. A similar finding was identified in Shin et al.’s study, where they tried to identify the predictors of morbidity and mortality in patients undergoing surgery for intestinal perforations. A p-value of 0.002 in their study for the significance of urea levels in predicting postoperative outcomes was discovered, further strengthening the argument for including urea levels at the time of admission in the POSSUM score [14]. Thus, a higher blood urea nitrogen (BUN) level predicts a higher risk of morbidity and mortality.

Statistical significance was also seen between malignancy and postoperative outcome in our cases of ileal perforation. Among the patients with malignancy, one survived and one succumbed postoperatively. Thus, it is clear that patients in whom ileal perforations occur, with underlying malignancy, have a higher risk of morbidity and mortality in the postoperative period. This can be attributed to the fact that all perforations are managed on an emergency basis, irrespective of the malignancy status of the patient. Thus, the underlying immunocompromised state more susceptible to complications dictates the outcome of surgery. Out of the remaining 38 patients in our study who did not have malignancies, six patients did not survive. A similar conclusion was seen in the study that was carried out by Otani et al., where they concluded that emergency surgeries in case of perforations have a generally higher risk of developing adverse postoperative outcomes. Furthermore, they add that the long-term outcome post surgery does not depend on the surgery per se, but on the underlying malignancy [15]. A similar sentiment was implied in Nakao et al.’s study whose conclusion was that metastatic colorectal carcinoma is the predictor for postoperative outcome and not necessarily the site of perforation [16]. Thus, it is logical to include malignancy status in the POSSUM scoring system and not the site of perforation.

Among the predictors used in the POSSUM scoring system, we found that cardiac signs, pulse, WBC count, Glasgow Coma Scale (GCS) score, sodium, and electrocardiogram (ECG), among the physiological parameters, did not have any statistical significance in relation to predicting postoperative outcome.

In our sample of 40 patients, only one patient was on cardiac drugs and that may be the reason for the calculated insignificance among the POSSUM parameters. Among other parameters within the ambit of cardiac signs, none of the patients presented with any of the signs or symptoms that qualify for inclusion in the POSSUM calculation. While most studies indicate that cardiac disorders play a major role in the prediction of postoperative outcomes, some studies, like the one carried out by Lee and Im, show that cardiac disease may also be statistically insignificant in the identification of possible adverse outcomes post surgery. In their study, patients who underwent surgery for gastrointestinal perforations were analysed and their underlying cardiac status was compared with postoperative outcomes. They found that the p-value was 0.982, indicating that it has no statistical significance in the postoperative 30-day mortality [17].

Multiple studies show that heart rate, although a crude predictor, can be used to predict postoperative outcomes. For example, the widely used Acute Physiology and Chronic Health Evaluation (APACHE) II score is calculated with heart rate being a variable to be considered. Our study showed that pulse may not play a significant role in the prediction of postoperative outcomes. The p-value calculated was found to be 0.78. Even in the study carried out by Shin et al., heart rate was found to be an insignificant predictor of postoperative mortality as the p-value was found to be 0.206 [14]. The use of preoperative heart rate in the prediction of postoperative mortality and morbidity may thus be acknowledged as only a parameter that indicates a more sinister underlying pathology which presents as a deranged heart rate. For example, while heart rate itself may not be a significant parameter, underlying sepsis or haemorrhage which alters the pulse of the patient may play a more significant role in postoperative predictions.

Similarly, the WBC count at the time of presentation was found to be statistically insignificant in the prediction of postoperative outcomes in this study. Along similar lines, the study undertaken by Shin et al. shows that the correlation between WBC count at the time of admission as a predictor of postoperative mortality within one month of surgery has a p-value of 0.892 [14]. According to Kudou et al., it is the neutrophil-to-lymphocyte ratio that serves as a predictor of postoperative outcomes, as it was found to be significantly affecting the postoperative period by being a risk factor for intra-abdominal abscesses and wound infections [18].

Our study also highlights the insignificant association of preoperative sodium levels with postoperative results. We found that the p-value was 0.262, which shows that the patients’ outcomes had no correlation to their sodium levels at the time of admission. A review of existing literature also throws light on similar findings in other studies. Mane et al.’s study on the evaluation of POSSUM also found that sodium was an insignificant contributor as a predictor, although it is a part of the calculated POSSUM score (p-value = 0.518) [19]. Another study carried out in Karad, Maharashtra, India, by Shiragave et al., found that only 13 out of their sample of 77 patients suffered from hyponatremia at the time of admission, accounting for approximately 17% of the study population [20]. The logical conclusion to this finding is that preoperative derangement of sodium levels does not appear to affect the postoperative outcome due to the rapid correction of the derangement both pre- and intraoperatively. For example, one of the safest intravenous fluids that is often given to patients in the emergency room at the time of presentation for fluid resuscitation is 0.9% normal saline, which in itself starts the correction of deranged sodium levels. In the emergency setting, following primary resuscitation protocols, large-bore intravenous access is attained and fluids are started at the earliest to prevent further deterioration. The fluids of choice for primary resuscitation, as shown in Ramesh et al.’s study, which analysed the best strategies of fluid resuscitation in trauma, are generally Ringer’s lactate solution or 0.9% normal saline, staying true to the guidelines that have been framed by the Advanced Trauma Life Support (ATLS) [21]. It appears that the speed at which the correction of sodium derangements is started negates its importance in the prediction of postoperative outcomes.

The correlation between ECG findings and the risk of adverse postoperative outcomes was also found to be statistically insignificant. Out of the sample population of 40, seven patients succumbed, although their ECGs were found to be within normal limits. Thus, the role of preoperative ECG being used as a predictor in the POSSUM calculation can be questioned. In line with this notion, we also found that there are multiple studies that show that ECG findings are statistically insignificant in the assimilation of postoperative outcomes. The p-value obtained for the same in Jha et al.’s study was found to be 0.689 [22] and in Mane et al.’s study, it was 0.132 [19], indicating that the inclusion of ECG as a parameter in the calculation of POSSUM may not be justified. According to POSSUM, any abnormality in ECG rhythm is given a score of eight in its physiological score [23]. From our study, and from the examples given, it is clear that ECG as a parameter in the POSSUM calculation may not be valid. The explanation for this may stem from the fact that it is a crude measure of cardiac activity and does not differentiate between underlying cardiac disease and cardiac abnormality that is being brought about by the acute disease that the patient is suffering from.

All patients included in our study presented with a GCS of 15, indicating that there was no statistically significant correlation that we could calculate to evaluate its validity in the POSSUM score or its effect on postoperative outcome. After an extensive literature review, we found that there were no studies that correlated the GCS of a patient at the time of presentation with their postoperative outcome in cases of ileal perforation. The study conducted by Kang et al. appears to be one of a few studies that compare GCS at the time of presentation with postoperative outcome but the cases included were those who suffered from trauma to the abdomen, not necessarily limited to perforations. In their study, a p-value of 0.805 was obtained and the calculation was made possible as they had patients who presented with GCS scores ranging from 10 to 15 [24]. Again, these findings bring into question the use of GCS as one of the parameters to calculate the POSSUM score. GCS is used widely for the neurological assessment of patients in an emergency situation and helps in the triage of patients. After calculating the patient’s GCS, management is aimed at improving it by correcting the underlying cause rapidly. In ileal perforation, the perforation itself serves as the underlying disease and its correction automatically improves GCS. In most cases, irrespective of GCS, ileal perforation patients undergo exploration and are treated surgically. Thus, the impact of GCS on the final outcome becomes questionable as there is no score of the GCS at which one would not perform a laparotomy for the patient.

Once each patient was assessed and their respective scores designated, we found that the mean POSSUM morbidity and mortality scores were 73.78 and 26.5, respectively. Using these scores and their counterparts after the calculation of scores for patients who were struck with adverse effects of surgeries or succumbed to surgeries, the receiver operating characteristic (ROC) curves were plotted and the area under the curve (AUC) obtained was 0.945 and 0.924, respectively, depicting that the POSSUM score is an excellent predictor of postoperative morbidity and mortality in ileal perforation patients irrespective of aetiology. Existing literature on POSSUM scoring is generally favourable but some studies have shown that it may not be completely reliable. In line with the findings of our study, the study conducted by Nachiappan and Litake identified ROC with AUC tending towards 0.99 in both mortality and morbidity scoring, reiterating that the score is an excellent predictor of postoperative adverse events [25]. A few studies like that conducted by Shekar et al. show the AUC obtained for both POSSUM morbidity and mortality calculations to be 0.66, demonstrating a moderately good predictive value on postoperative outcomes [23]. On further assimilation of the data collected, we identified the AUC derived from the ROC constructed for P-POSSUM mortality to be 0.966, showing that the P-POSSUM variation continues to be an excellent predictor of postoperative mortality.

It must be noted that an excellent predictor of morbidity or mortality does not necessarily mean an accurate predictor. Through multiple studies on assimilating the accuracy of the POSSUM score, it is widely recognized that the POSSUM score overestimates morbidity and mortality. Nachiappan and Litake’s article categorically points out that the POSSUM score over-predicts mortality [25]. Our study also identified similar findings where, on calculation, the predicted morbidity and mortality were higher than the actual number of patients with adverse postoperative outcomes. The expected number of deaths was calculated to be 19 whereas the observed deaths were found to number only seven. Similarly, the number of patients who were going to suffer from complications was predicted to be 22 but the actual number of patients who suffered postoperative complications was eight. We also identified that the observed vs. expected mortality rates in ileal perforation patients did not have much of a difference while calculating these rates using the P-POSSUM variation; 12 expected vs. eight observed mortalities. This is also in line with existing literature on the topic, as succinctly put by Mane et al., the P-POSSUM has a better correlation between observed and expected mortality rates making it a better score to use for the prediction of the same in postoperative patients [19].

## Conclusions

Scoring systems are an indispensable tool to plan an appropriate line of management for any surgical pathology. To that end, there exist multiple scoring systems, of prominence being the POSSUM and P-POSSUM scores. Certain parameters used to calculate the POSSUM and P-POSSUM scores appear to bear no statistical significance to the final score, highlighting that these need to be revisited and modified to enhance the ease of calculation by hospital staff at the time of admission.

Overall, the predictive power of POSSUM and P-POSSUM is valid although they may overestimate morbidity and mortality in patients suffering from ileal perforation, irrespective of aetiology. All factors considered, they appear to be good stratification tools not only for surgical risk but also for patient counselling. Considering that the parameters of calculation are easy to identify and calculate, an argument can be made for the continued use of these scores in resource-deficient settings like ours and during emergency situations when a decision regarding surgical management is to be taken. Applying these scores in such settings, the ideal and exact situation that they were developed for must be taken as crucial for their validation by taking into account the findings that have been made in this study.
